# Geographic patterns of poor HIV/AIDS care continuum in District of Columbia

**DOI:** 10.1186/s12981-018-0189-8

**Published:** 2018-01-24

**Authors:** Suparna Das, Jenevieve Opoku, Michael Kharfen, Adam Allston

**Affiliations:** 1 Strategic Information Division, HIV/AIDS, Hepatitis, STD and TB Administration (HAHSTA), District of Columbia Department of Health, Government of the District of Columbia, 899 North Capitol St., NE/Fourth Floor, Washington, DC, 20002 USA; 2 HIV/AIDS, Hepatitis, STD & TB Administration (HAHSTA), District of Columbia Department of Health, Government of the District of Columbia, 899 N. Capitol St., NE, Washington, DC, 20002 USA

**Keywords:** HIV care continuum, Bayesian relative risk, Disease mapping, District of Columbia, Area based prevention intervention strategies

## Abstract

**Background:**

Concurrent with the UNAIDS 90-90-90 and NHAS plans, the District of Columbia (DC) launched its 90/90/90/50 plan (*Plan)* in 2015. The *Plan* proposes that by 2020, 90% of all DC residents will know their HIV status; 90% of residents living with HIV will be in sustained treatment; 90% of those in treatment will reach “Viral Suppression” and DC will achieve 50% reduction of new HIV cases. To achieve these goals targeted prevention strategies are imperative for areas where the relative risk (RR) of not being linked to care (NL), not retained in any care (NRC) and low viral suppression (NVSP) are highest in the District. These outcomes are denoted in this study as *poor outcomes of HIV care continuum*. This study applies the Bayesian model for RR for area specific random effects to identify the census tracts with poor HIV care continuum outcomes for DC.

**Methods:**

This analysis was conducted using cases diagnosed from 2010 to 2015 and reported to the surveillance system from the District of Columbia Department of Health (DC DOH), HIV/AIDS, Hepatitis, STD and TB Administration. The jurisdictions of the District of Columbia is divided into 179 census tracts. It is challenging to plot sparse data in ‘small’ local administrative areas, characteristically which may have a single-count datum for each geographic area. Bayesian methods overcome this problem by assimilating prior information to the underlying RR, making the predicted RR estimates robust.

**Results:**

The RR of NL is higher in 59 (33%) out of 179 census tracts in DC. The RR of NRC was high in 46 (26%) of the census tracts while 52 census tracts (29%) show a high risk of having NVSP among its residents. This study also identifies clear correlated heterogeneity or clustering is evident in the northern tracts of the district.

**Conclusion:**

The study finds census tracts with higher RR of poor linkage to care outcomes in the District. These results will inform the *Plan* which aims to increase targeted testing leading to early initiation of antiretroviral therapy. The uniqueness of this study lies in its translational scope where surveillance data can be used to inform local public health programs and enhance the quality of health for the people with HIV.

**Electronic supplementary material:**

The online version of this article (10.1186/s12981-018-0189-8) contains supplementary material, which is available to authorized users.

## Background

The HIV care continuum is an internationally-recognized framework, accepted in the United States as a series of stages from the time an individual receives a diagnosis of HIV through the successful treatment of their infection with HIV medications [1, 2]. The care continuum was introduced as the HIV treatment cascade in 2010 as a part of the United States National HIV/AIDS Strategy (NHAS). It proposes being in care not only leads to better health outcomes for people living with HIV, but it may also help reduce new transmissions within the community. Thus evaluating HIV care dynamics is a vital step to gauging the strengths of HIV programs and addressing the gaps in the care continuum [3]. Effective HIV treatment as prevention requires people be tested, know their HIV status, be linked and retained in care, and achieve viral suppression through effective antiretroviral therapy (ART) [4, 5]. Treatment as prevention (TasP) is an HIV prevention intervention where treating an HIV-positive person with ART is used to diminish the potential of HIV transmission to the negative partner [6]. The core strategy of treatment as prevention in the context of the care continuum is to make ART available to the people. Early introduction of ART for people living with HIV (PLWH) is critical in ensuring better individual health outcomes and reducing HIV transmission [7, 8].

In 2016, concurrent with the UNAIDS 90-90-90 and NHAS plan, the District of Columbia (DC) launched its 90/90/90/50 plan (*Plan*). This *Plan* proposes that by 2020, 90% of all DC residents will know their HIV status; 90% of residents living with HIV will be in sustained treatment; 90% of those in treatment will reach “Viral Suppression”; and DC will achieve a 50% reduction in new HIV cases. One of the first significant steps to achieving these goals is to identify geographic areas with higher levels of relative risk (RR) of poor HIV care continuum outcomes. Areas of poor care continuum outcomes where the resident population is at greater risk of *not being linked to any care* (NL) or *not retained in any* care (NRC) or *have lower levels or no viral suppression* (NVSP).

Mapping health outcomes and detecting geographic disparities in care are valuable tools in public health, helping decision-makers to recognize areas which requires resources [9]. Local as well as federal agencies have used surveillance data to analyze the care continuum by demographic characteristics and HIV transmission risk [7, 10]. Studies have been conducted to understand spatial patterns along the HIV care continuum [11] and the feasibility of using the HIV care continuum to identify geographic areas at most risk for HIV and poor health outcomes [12].

There are different models and methods to develop maps of diseases including simple statistical illustration, informal methods, basic models, multilevel models etc. This study aims to identify areas of poor HIV care continuum outcomes in DC using Bayesian disease mapping methods. Disease mapping using Bayesian approach consists of prior information about variation in disease rates, in addition to observed events in each area. It also considers the spatial pattern of disease in which close geographic areas have more similar disease rates [13–16].

The results from this analysis will be used by DC Department of Health (DOH) to shape its HIV prevention and care programs as well as used to implement the targets mentioned in the 90/90/90/50 plan. Apart from the immediate impact of the study in shaping DC’s care continuum, the study is also significant as it uses local surveillance data to identify areas of risks of being out of care continuum thus not relying on national estimates.

## Methods and data

Disease mapping comprises of a set of statistical techniques, which assist in generating accurate maps based on estimations of incidence, prevalence, and mortality of disease [17]. Contemporary disease mapping, the cornerstone of spatial epidemiology, identifies geographical risk disparities and clustering within small areas. This is commonly called small-area disease or health mapping [18, 19]. The crucial question remains whether the patterns, variations, and clusters observed from the disease map are significant or simply variations due to small samples [19]. Spatial analysis and disease mapping of the HIV care continuum has been used to identify areas of poor outcomes as a tool for identifying target areas for prevention and intervention strategies [11, 20]. The principal limitation of using these traditional measures is the uneven distribution of the population among tracts [21]. This may lead to inflated rates yielding erroneous and inaccurate disease maps and risk identification. Another challenge in disease mapping is plotting sparse data in ‘small’ local administrative areas, which, characteristically, may have a single-count datum for each tract [18, 19]. These challenges are usually met by using standard mortality rate (SMR) for disease mapping. SMR is the ratio of between observed number of events in a study population and the expected number of events in the study population. Unfortunately, SMRs also suffers from several drawbacks [22]. The ratio estimators can yield large variations in approximation with comparatively minor changes in expected value [22, 23]. The SMR is essentially a saturated estimate of relative risk and hence is not parsimonious. SMR is a crude estimate and can provide unsteady estimations owing to its ratio formula, and does not consider the correlation among neighbors [24, 25].

Bayesian approach to disease mapping overcomes these drawbacks. Relative risk (RR) of disease in area *i* was shown as $\theta_{i}$. Clayton and Kaldor [16] suggested a method (Besag, Mollie and York—BYM) that models the RR as a function of spatially structured and unstructured random effects [23]. The unstructured random effects are modeled on a Conditional AutoRegressive-prior or CAR-prior. These CAR models are frequently used both by statisticians and epidemiologists, and their application is enabled by existing software such as OpenBUGS [26]. The central feature of all these models is to deliver some shrinkage and spatial smoothing of the raw RR approximations that otherwise would be calculated individually for each area. Such shrinkage provides a further steady approximation of the pattern of underlying risk than that delivered by the raw estimates. The pattern of the raw risks, based on the size of the population at risk, leads to a noisy and fuzzy image of the true unobserved risks [27].

This paper uses a model for relative risks for area specific random effects which are deconstructed into a component that models the effects that vary in a structured manner in space (clustering or correlated heterogeneity) and a component that models the effects that vary in an unstructured way between areas (uncorrelated heterogeneity) [22]. The parameter of interest is θ_*i*_, the RR that quantifies whether the area i has a higher (θ_*i*_ > 1) or lower (θ_*i*_ < 1) occurrence of cases than that expected from the background population.

The model was introduced by Clayton and Kaldor and extended by Besag, York, and Mollie, [16, 28] and its formulation is described as:

$$y_{i} = poisson\left( {E_{i} \theta_{i} } \right)$$

$$\log \left( {\theta_{i} } \right) = \alpha + u_{i} + v_{i} ,$$

where *α*: trend component that is overall level of relative risk. *u*_*i*_: [(spatial overdispersion) spatial correlated heterogeneity]: It is logical that the close areas have similar relative risks. To take this similarities into account, the random variable *u*_*i*_ which is uncorrelated with other (*u*_*j*_) included in the model. For this component, spatial correlation structure is used where estimates relative risk in each area are dependent on adjacent areas. The conditional autoregressive model proposed by Besag et al. is:

$$\left[ {u_{i} |u_{j} ,\;{\text{i}} \ne {\text{j}},\tau_{v}^{2} } \right] \sim N\left( {\overline{\text{u}}_{i} ,\tau_{v}^{2} } \right),$$

where $$\overline{{{\text{u}}_{i} }} = \frac{1}{{\sum {w_{ij} } }}\sum {u_{i} w_{ij} } ,$$$$\tau_{v}^{2} = \frac{1}{{\mathop \sum \nolimits_{j} w_{ij} }},$$*w*_*ij*_ = 1 if *i, j* are adjacent (or 0 if they are not),*v*_*i*_: [Non-Spatial over dispersion (spatial uncorrelated heterogeneity)]: By the formulation of the model for spatially correlated heterogeneities, the variance is dependent on the number of neighbors and independence couldn’t be defined well. In order to justify this problem, another component (*v*_*i*_) is introduced that is an uncorrelated over dispersion parameter. The prior distribution of this parameter is $$v_{i} \sim N\left( {0, \tau_{v}^{2} } \right)$$.

Parameters *τ*_*v*_^2^ and *τ*_*u*_^2^ control the variability of *v* and *u* [22] respectively. In a full Bayesian analysis, prior distributions must be specified for those parameters (Additional file 1: Figure S1). We considered gamma prior distribution for both *τ*_*v*_^2^ and *τ*_*u*_^2^, as suggested by Bernardinelli [29] (Additional file 1: Figure S1). The OpenBUGS Code used in the analysis was borrowed from Lawson [22]. Model fitting was done using two separate chains starting with different initial values. Convergence was checked by visual examination of time series plots of samples for each chain and by computing the Gelman and Rubin diagnostic [30]. The first 5000 samples were discarded as a burn-in; each chain run for a further 75,000 iterations for all the models [31]. Results were mapped using Maptitude software. To investigate how the models fit the data well, a deviance information criterion (DIC) was applied.

**Fig. 1:**
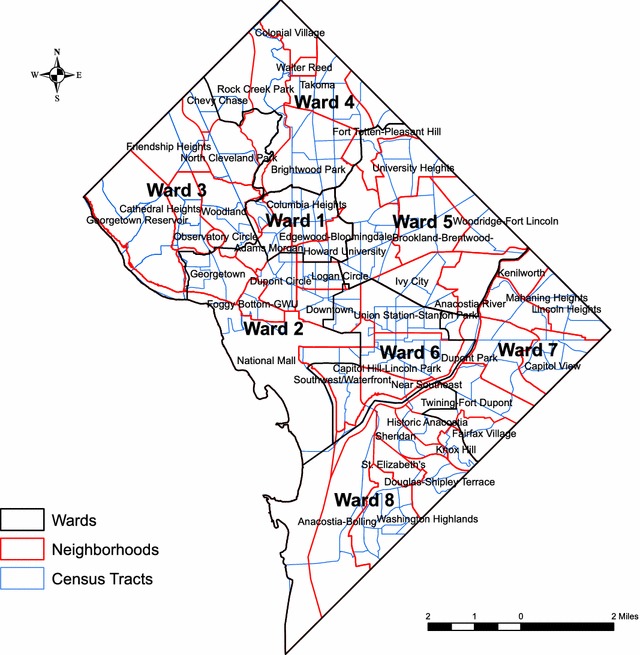
The boundary map of District of Columbia, delineating Wards, Neighbourhoods and Census Tracts

Data analyzed for the study were obtained from the Enhanced HIV/AIDS Reporting System (EHARS) of the District of Columbia Department of Health (DC DOH), HIV/AIDS, Hepatitis, STD and TB Administration (HAHSTA) for years 2010–2015. The variables were extracted using SAS code from persons and laboratory dataset of EHARS. For those cases NL (Not linked to care) outcome, the cases were based on the residence of diagnosis. NRC (Not retained in any care) outcome cases were based on the current address of the resident, cases with no laboratory information on being in any care after being linked were treated not retained in care outcome. For NVSP (Not Virally Suppressed) current addresses were geocoded; cases not suppressed or no viral load reported were treated as not virally suppressed. The geographic coordinates associated with each case of infection were assigned using Maptitude Geographic Information System (GIS) software. Between 2010 and 2015 there were 1125 eligible cases with an available address that could be geocoded. Post Office Box numbers, DC Detention centers, and homeless were not included in the analysis to avoid spatial bias. Overall, the District of Columbia comprises 179 census tracts, 8 wards and 49 neighborhoods (Fig. 1 boundary map of District of Columbia). The cartographic boundary map is based on the 2010 US census.

The following table (Table 1) have the socio-economic and demographic variables of people in the wards of DC. The data is obtained from Office of Planning, Government of District of Columbia. Ward six (84,290) has the highest number of people and Ward seven (73,290) has the lowest number of people. The district shows racial variation in distribution, with highest percent of black population is ward one (95.20%) while white population is highest in ward three (8.50%). Unemployment rates are highest in ward eight (12.70%) and lowest in ward three (2.60%). High school education does not show significant variation across the wards, with lowest in wards seven and eight (83.0%).

**Table 1:** Social, economic and demographic characteristics of wards in District of Columbia

Variables	Ward 1	Ward 2	Ward 3	Ward 4	Ward 5	Ward 6	Ward 7	Ward 8
Total population^a^	82,859	77,645	83,152	83,066	82,049	84,290	73,290	81,133
Male^a^	49.90	50.5	44.7	48.60	47.20	47.90	46.30	43.80
Female^a^	50.10	49.50	55.30	51.40	52.80	52.10	53.70	56.20
White^a^	56.70	77.20	83.90	28.40	22.10	58.00	3.00	5.40
Black and African American^a^	31.50	9.80	8.50	57.90	71.90	36.80	95.20	93.60
Unemployment rate^a^	5.10	2.70	2.60	6.90	9.00	4.70	11	12.70
High school education^a^	87.7	94.90	97.60	87.10	86.30	92.60	83.0	83.0

^a^ Office of planning, District of Columbia

Table 2 defines the HIV care continuum measures followed and applied in the District of Columbia. The ultimate goal of HIV treatment is to achieve viral suppression, meaning the amount of HIV in the body is very low or undetectable. This is important for people with HIV to stay healthy, live longer and reduce their chances of passing HIV to others.

**Table 2:** Care continuum outcome measures followed in DC

Measure	Definition	Levels
HIV cases living in DC	Number of cases diagnosed with HIV through 2015 and presumed living in DC at the end of 2016	
Linkage to care	Evidence of diagnosis date to first CD4 and/or viral load laboratory	Living in DC; any evidence of a CD4 and/or viral load after initial lab in DC Newly diagnosed: any evidence of a CD4 and/or viral load lab within 3 months initial HIV diagnosis
Retained in care	Stability of care in 2016	Two viral load and/or CD4 labs reported more than 90 days apart in the year Out of care: No lab reported in the year
Virally suppressed	Suppression any time after HIV disease diagnosis	Suppressed: reported viral load ≤ 200copies/ml Not suppressed: reported viral load ≥ 200 copies/ml No viral load reported

This study used Bayesian spatial statistics to find areas of poor outcomes along the HIV care continuum. This study used three measures of the care dynamics that were defined as poor outcomes of HIV care continuum: (1) not linked to care—diagnosed in District of Columbia, but do not have any evidence of a CD4 and/or viral load after initial lab in DC. For newly diagnosed, no evidence of a CD4 and/or viral load lab within 3 months of initial HIV diagnosis. (2) Not retained in care—comprises of those who have no lab reported within year of analysis or do not have two labs reported more than 90 days apart within the year of analysis. (3) Not achieving viral suppression—comprises those who have not achieved viral suppression and those with no viral load reported within the year analysis.

## Results

The results compares three forms of disease mapping for HIV care continuum measures for the DC DOH, HAHSTA conducted an internal evaluation of the percentage of DC residents with undiagnosed HIV infection [32]. The analysis found 9–14% of the people in the District are unaware of their HIV diagnosis [33].

### Not linked in care (NL)

**Fig. 2:**
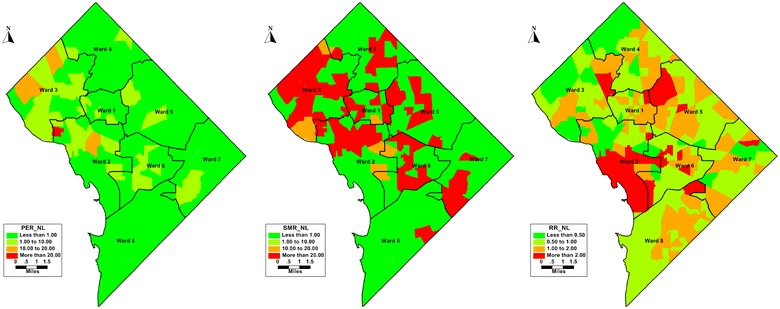
Maps of simple rates (left), raw SMRs (middle) of *not linked to HIV care* compared to smoothed Relative Risks from Poisson log normal model (right) in District of Columbia (2010–2015)

Figure 2 (Not linked to care) shows disease mapping for people not linked to any care in the census tracts of the District. The simple rates, which is the percent of cases with no lab report by the total population. Two tracts in wards three and a single tract in ward 1 with more than 10% of NL, compared to the SMR map which has higher values across in all tracts except in ward eight. Tracts with SMR greater than 1.0 indicates not linked to HIV care compared to what was expected. The *θ*_*i*_ (RR) of NL is higher than 1 in 59 (33%) out of 179 census tracts in DC. *θ*_*i*_ map shows a smoother version of SMR in the Bayesian estimates showing parts of ward two, three, five and six with highest risk of not being linked to any care. The idea is that the smoothed estimate for each tract borrows strength from the data in the surrounding tracts making the results stable.

**Fig. 3:**
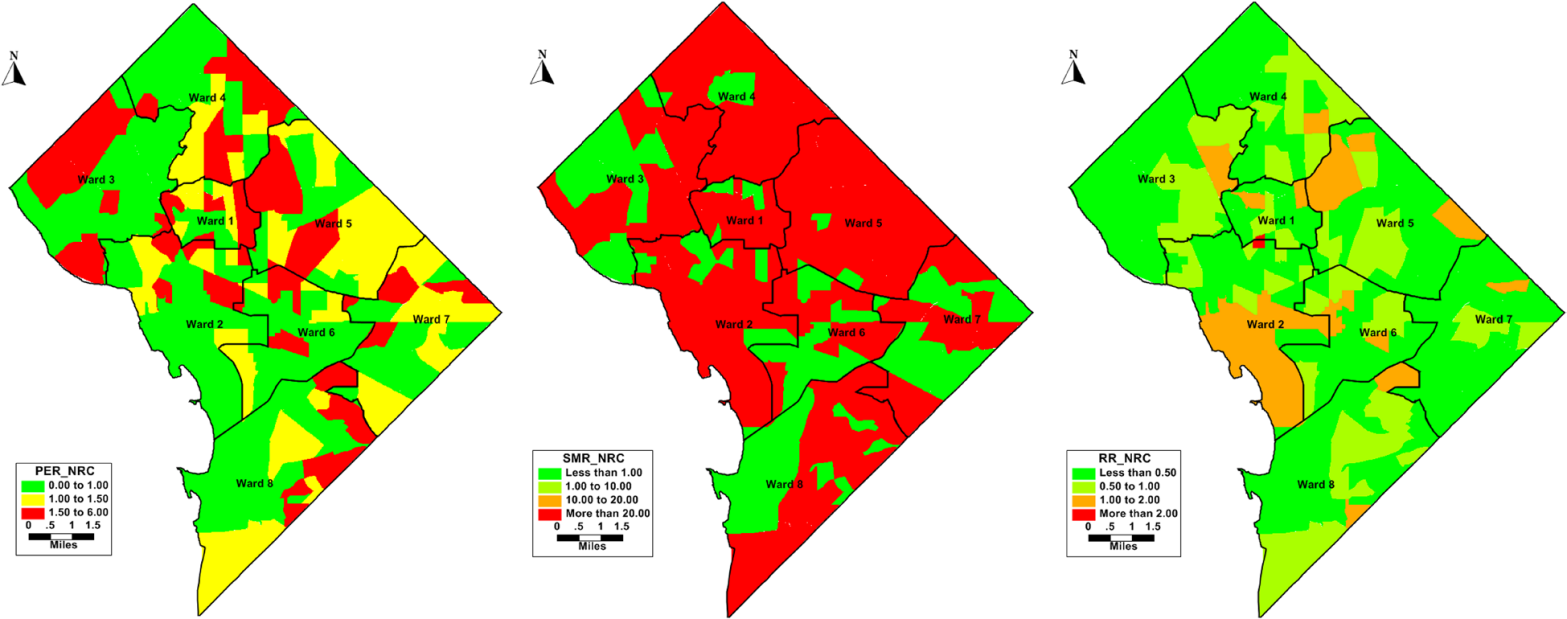
Maps of simple rates (left), raw SMRs (left) of *not retained in any care* compared to smoothed Relative Risks from Poisson log normal model (right) in District of Columbia (2010–2015)

Figure 3 (NRC) shows disease mapping for people NRC in the census tracts of the District. The higher rates of NRC is evident in almost all the wards. For SMR most tracts have SMR more than 20. $\theta_{i} > 1$ for NRC is evident in 13 (7%) of the 179 census tracts. Compared to SMR disease map, $\theta_{i}$ maps shows smoothed results with RR more than in few tracts across DC.

### Not virally suppressed (NVSP)

**Fig. 4:**
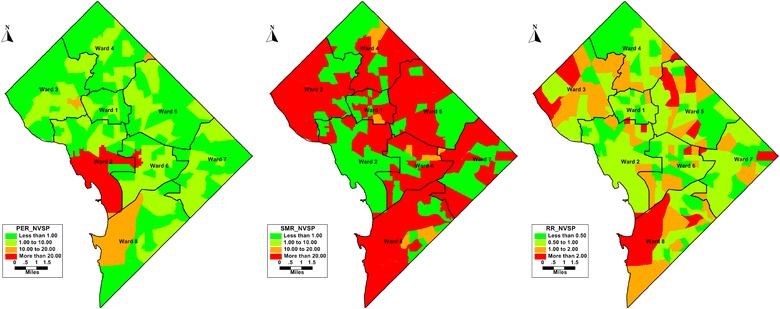
Maps of simple rates (left), raw SMRs (left) of *not retained in any care* compared to smoothed Relative Risks from Poisson log normal model (right) in District of Columbia (2010–2015)

Figure 4 show simple rates mapping where the highest rates of NVSP are in the tracts of Wards two and six. SMR with greater than 1.00 are most prevalent in the tracts of Wards three, five, six and eight. *θ*_*i*_ (RR) of NVSP is more than 1 in 52 (29%) of the 179 census tracts in the DC by the end of the study period. Bayesian estimates of *θ*_*i*_ display a smoother map compared to SMR, tracts in wards three, five and eight have *θ*_*i*_ more than 1.

Table 3 shows α (intercept) which is the overall level of RR poor outcomes in DC of NL (− 0.02389), NRC (− 0.3182) and NVSP (− 0.4891). The table also shows the posterior mean and 95% credible intervals of the variance components for the model. The variability of the relative risk is attributed more to the uncorrelated heterogeneity than to the spatial structure effects. The *τ*_*u*_^2^ for the NL is 34.21 vs. 233.4 for the NRC model, are substantial number indicating that the risk in any given tract in DC does not have any similarities to that of the neighboring tracts. Comparatively, the *τ*_*u*_^2^ for NVSP is smaller than the preceding values at 0.8418; thus the risk of NVSP in any given tract in DC may be similar to that in the neighboring areas. The DIC is low for all the models, but lowest for the NL.

**Table 3:** Posterior statistics of the variance components and model

Not linked to care				Not retained to care				Not virally suppressed			
	Mean	STD dev	Credible interval		Mean	STD dev	Credible interval		Mean	STD dev	Credible interval
α	− 0.2389	0.09904	− 0.4424, − 0.05504	α	− 0.3182	0.09017	− 0.5017, -0.1474	α	− 0.4891	0.096	− 0.6847, − 0.3074
$\tau_{u}^{2}$	34.21	247.8	0.3736, 276.1	$\tau_{u}^{2}$	233.4	790.1	0.9515, 2544	$\tau_{u}^{2}$	0.8418	1.429	0.2251, 3.847
$\tau_{v}^{2}$	639.7	1231	1.369, 4364	$\tau_{v}^{2}$	1.578	0.4645	0.9697, 2.708	$\tau_{v}^{2}$	541.2	1192	0.9033, 4113
DIC			442.8	DIC			664.7	DIC			484.7
PD			21.78	PD			80.63	PD			− 28.41

**Fig. 5:**
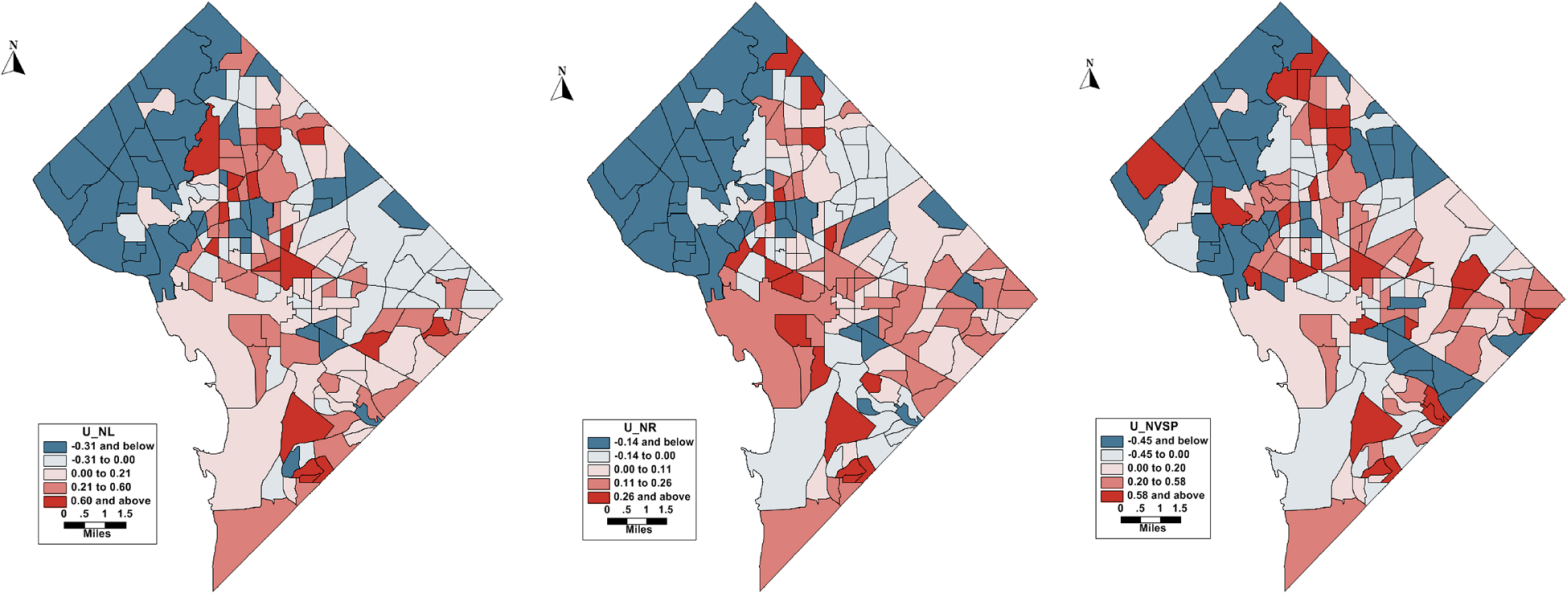
Posterior expectation of u (correlated heterogeneity) for the Bayesian model

For NVSP results in the spatial blocks with low values are evident in the north as well as in the south, but a pattern of spatial association is not as clear as in the previous two outcomes, which the low *τ*_*u*_^2^ results. The *τ*_*u*_^2^ is large for NL and NRC outcomes, indicating areas of correlated heterogeneity. Figure 5 maps areas of correlated heterogeneity over space in the district, which displays blocks of values for all the three models. The resulting correlated heterogeneity is the random effect that arises from a model where the spatial unit is correlated with neighboring spatial units. This denotes spatial autocorrelation of the outcomes among the census tracts.

## Discussion

This study used surveillance data to identify geographic patterns of the areas of poor adherence to the HIV care continuum for people living with HIV in DC. The smoothed Bayesian estimate for each ‘borrow strength’ (precision) from the data in the surrounding areas which takes into account a spatial pattern of the outcome (i.e. Tobler’s First Law of Geography, things that are nearby are more related than things that are far away). From the results it is evident that percentages (will be addressed as rates) used to identify the areas of lower care continuum outcome is specially unstable as much of the variation in the rates is attributable to the statistical noise due to the small number in low-population tracts [24]. Traditional standard mortality rates calculated simply as a ratio of observed and expected is another approach that is widely used by epidemiologists. There are shortcomings [34] in SMR which is calculated as the following: $SMR = \frac{Observerd}{Expected}$. The results produced through the SMR are not robust enough to be used for policy implementation particularly in DC which calls for small area estimation. The results in accordance with Clayton and Kaldor’s methods are smoothed for areas of poor HIV care continuum outcomes. The smoothed estimates points the tracts where people are at higher risk of being not linked to care, not retained in care and not virally suppressed. The statistical noise which rates and SMRs suffer from is smoothed in the bayesian results.

Tracts in wards two, three, five and six have show the highest risk for people being not linked to care (NL). The total number of tracts of NL with *θ*_*i*_ > 1 was 59 (33%) of the 179 tracts. The results will help DOH shape its programs to the areas with higher risk. The lower linkage areas would be monitored and evaluated on the factors of the suboptimal performance to inform and design an enhanced comprehensive linkage service system. Upon diagnosis, it is critical for patients to be linked with medical treatment and anti-retroviral therapy in order for them to stay healthy. Timely linkage to care is a significant focus of DOH’s Plan. This would involve area based expansion of access to treatment and related services which also includes AIDS Drug Assistance Program (ADAP) as well as other medical assitance programs.

Results show the tracts across DC where the risk of retention to care (NRC) are higher. The total number tracts where the risk of NRC is θ_*i*_ > 1 is only 13 (7%). The lower retained areas would guide strategies in identifying and recapture people living with HIV who have been out of care. Though low in number yet DOH would target these areas to offer facilities in order to assist people to remain in care. It is important for the linked and diagnosed individuals to be retained in case as drug therapy can improve health outcomes and lowers transmission risk.

There are 52 tracts where the residents are at higher risk (θ_*i*_ > 1) of not achieving viral suppression. DOH will use the results to inform its strategies for ending HIV epidemic in the district. DOH is currently working with providers to remove barriers to care and adhering in care. In 2015, Whitman-Walker Health launched the Mobile Outreach Retention and Engagement (MORE) initiative, supported by funding from DOH and the Washington AIDS partnership [33]. The MORE mobile medical team would provide evaluation, lab tests, and counseling services in patients’ home and at pop-up locations in the community. The areas would help MORE to strategize where pop-up clinics could be most helpful. In the areas of lower viral suppression DOH would work with the pharmacies in the areas to improve access to prescriptions and track medication treatment adherence as well as promote telemedicine approaches for adherence support. These results would also be used to find areas to support the *Plan’s* approach where DOH would partner with other DC government agencies to address the social support needs of the person living with HIV in the District.

DOH will also be able to evaluate the barriers to be linked to care in the census tracts where the risk of NL is high. Under the *Plan* DC is targeting to implement several programs that would help end the HIV epidemic in the district. Those programs include targeted expansion of access to treatment and related services.

## Conclusion

The analysis identified the areas for District of Columbia’s 90/90/90/50 plan which will be used to target testing activities and care activities of the DC DOH. The results also show clustering of the outcomes of HIV care continuum. The exact reason for clustering is not addressed in the study. Clustering may naturally be present in the are or may also stem from the presence of undetected ecological or “frailty effects” [22], which provides a scope for further exploration.

There are a few limitations to this study. The results are based on surveillance data reported by providers and laboratories to the HAHSTA. Therefore data for patients not reported to HAHSTA or those that moved out of the jurisdiction were not included in the analysis. Those addresses which could not be geocoded were not included in the analysis. Further it needs to be evaluated in social-determinants of health have any impact on HIV care continuum measures across space and time in DC.

## Additional file

**Additional file 1: Figure S1.** OpenBugs Code and prior distributions used in the model.
