# Supplementary material for: Geographic patterns of poor HIV/AIDS care continuum in District of Columbia
Source: AIDS Res Ther. 2018 Jan 24;15:2. doi: 10.1186/s12981-018-0189-8 (PMC5784661; doi:10.1186/s12981-018-0189-8)
Supplement: Supplementary file 1 — Additional file 1: Figure S1. OpenBugs Code and prior distributions used in the model. [file 12981_2018_189_MOESM1_ESM.docx]

**Additional file 1: Figure S1 OpenBugs Code and the prior distributions used in the model.**

MODEL

model

{

for (i in 1:m)

{

# Poisson likelihood for observed counts

y[i]~dpois(mu[i])

log(mu[i])<-log(e[i])+alpha+v[i]+u[i]

# Relative Risk

theta[i]<-exp(alpha+v[i]+u[i])

res_theta[i]<-exp(alpha+v[i]+u[i])

# Posterior probability of RR[i]>1

PP[i]<-step(theta[i]-1+eps)

res_PP[i]<-step(res_theta[i]-1+eps)

# Prior distribution for the uncorrelated heterogeneity

v[i]~dnorm(0,tau.v)

# Relative Risk decomposition

RR_het[i]<-exp(v[i])

RR_clust[i]<-exp(u[i])

}

eps<-1.0E-6

# CAR prior distribution for spatial correlated heterogeneity

u[1:m]~car.normal(adj[],weights[],num[],tau.u)

# Weights

for(k in 1:sumNumNeig)

{

weights[k]<-1

}

# Improper prior distribution for the mean relative risk in the study region

alpha~dflat()

mean<-exp(alpha)

# Hyperprior distributions on inverse variance parameter of random effects

tau.u~dgamma(0.5,0.0005)

tau.v~dgamma(0.5,0.0005)

}
